# The Cure of Epithelioma

**Published:** 1892-02

**Authors:** W. S. Gottheil

**Affiliations:** 25 West 53d St., New York City


### THE CURE OF EPITHELIOMA.

W. S. GOTTHEIL, M. D., NEW YORK.

[*Concluded.*]

4. *Escharotics.* These remedies for cancers of the skin have long lain under a cloud undeservedly. The advances of modern surgery have cast into obscurity methods that deserved a better fate. Thus they have fallen into the hands of irregular practitioners, have been advertised, and finally have fallen into such disrepute as to lead to their entire neglect.

Nevertheless, for the great majority of cancers of the skin, and with the exceptions mentioned in the preceding paragraphs escharotics form the best, least painful, most thorough, and most generally applicable form of treatment. The purpose for which they are used is often misunderstood: It is not so much the absolute destruction of the tissue in contact with the escharotic that is desired; that layer cannot but be superficial, and could be destroyed in other ways. It is the intense inflammation under and around the destroyed area that does the work. All new tissue is less highly organized than normal tissue, all new tissue breaks down under stimuli which normal tissue resists. Hence we aim to establish, if possible, an inflammatory process of just such intensity as to be beyond the endurance of the newly proliferated epithelial cells, and yet is within the bounds of tolerance of the maturer, fully developed normal tissues.

A variety of agents are employed for that purpose, some of which do not by any means fulfill the indications set forth above. Such far-reaching and intense escharotics as pure nitric acid and the acid nitrate of mercury and chloride of zinc defeat the very objects for which the remedy is used. They destroy the normal as well as the abnormal tissue; it is impossible to limit their action with any degree of accuracy, they are excessively painful, and they cause an entirely needless amount of deformity. On the other hand nitrate of silver and pyrogallol are hardly more than very mild caustics in whatever form they are applied; they may destroy a very superficial layer fully, but they act as gentle irritants to the deeper tissues and simply stimulate them to accelerated growth.

But in arsenic we have an agent at our command that exactly fulfills the requirements of the case. It can be used in various degrees of concentration, in accordance with the degree of inflammatory action which we desire to set up. It is not poisonous—the inflammation it sets up precluding all possibility of absorption. It is thoroughly manageable, and the amount of pain its application causes is readily borne in almost every case.

It has its limitations as before said. It cannot be used on cancers situated in the immediate proximity of the mucous orifices. Here one of the other methods of eradication must be employed. But wherever the situation is such as to permit its use, it is needless to try any of the others.

The mode of application is as follows: A fresh and stiff paste is made by mixing white arsenious acid and mucilage of acacia. The proportion of the two ingredients varies in different cases. I generally use equal parts of each, which makes a paste of about the right consistency. If the patient is very sensitive, it may be necessary to use one part of arsenic to two or four of acacia. The strongest paste is the best; the more intense the inflammatory action, the more positive the assurance of the impossibility of absorption. I have never, however, seen any symptoms of arsenical intoxication with any of them.

A curetting may or may not have previously been done. It is advisable in cases where the growth is fungating and projects much above the level of the skin, or where the amount of tissue to be destroyed is large. The escharotic acts much better upon an abraded surface.

The paste is now spread thickly and evenly upon a piece of linen, the area covered extending at least half an inch beyond the apparent confines of the growth.

There need be no fear of destroying healthy skin. Healthy skin reacts upon the escharotic and inflames; neoplastic tissue breaks down and dies. A much larger extent of tissue is always destroyed than is apparently affected; but this is due to the fact that the epithelioma extends further than is visible externally. And it is this faculty of the arsenic, to set up an inflammation of such intensity as to cause the breaking down of all the new tissue, that renders it especially valuable to us.

The plaster being put on, it is secured in place by rubber plaster, and the whole covered with absorbent cotton and a bandage.

The pain does not usually commence until a number of hours after the first application. As a rule it is very bearable and does not disturb the patient's sleep. There are cases, however, especially in hyperæsthetic females, when it becomes necessary to give an opiate. A small dose of morphine, 1 8 to 1-4 grain by the stomach at bedtime, is all that is required.

After the first few hours the parts in contact with the arsenic inflame and swell up, and the œdema may spread over a considerable territory. The eyelids may be swollen until the eye is almost closed; the lips and ear may be considerably tumefied. This always alarms the patient, and it is well to warn him of it beforehand. It occurs in consequence of the proximity of the inflammation set up to organs with loose subcutaneous connective tissue meshes which hold a good deal of serum. It is of no importance and gradually subsides.

When the plaster is first removed after twelve hours, the skin around the epithelioma is red, swollen, and angry looking, whilst the growth itself has assumed a slightly dusky appearance, marked largely however, by the white paste sticking to it. The part should be carefully cleansed with cotton and creolinised warm water, and a fresh application made of the paste.

This should be continued twice a day for a time which depends on the amount of tissue to be destroyed. In very superficial and circumscribed growths, one or two applications may be enough. In others it must be continued for three or four days.

When the destruction is complete, the new growth has turned dark brownish or black in color, and is not sensitive to the touch. It is dead.

The use of the paste is now stopped, and a flax-seed poultice is applied. Your object now is to promote the reactive inflammation of the healthy skin which is in contact with the dead tissue. The more frequently the poultices are renewed, and the better they are kept, the sooner will the necrotic mass be cast off.

After a few hours of poulticing, this yellow line of suppuration will become apparent at the edge of the normal skin. This deepens and broadens, and before long free suppuration is established. The suppuration surface spreads under the mass, and it is gradually cast off as a whole or by piecemeal. We can frequently help the casting off process by a skilful use of forceps and curved scissors.

The length of time that the poultices are required, depends on the mass to be cast off. As a rule two days are sufficient, but I have seen it take eight, in an exceptionally extensive case. There is, of course, no pain after the plaster is discontinued, and the patient does not object at all to the prolonged poulticing.

Finally the last fragment of necrotic tissue has come away, and we have in the place of our epithelioma an open ulcer of larger extent than the original growth itself. It is covered with an abundant layer of healthy pus, and already in those parts where the dead material was first cast off, red granulations are springing up abundantly. The question which now confronts us is: "Have we destroyed all the carcinomatous tissue, and is it safe to let the ulcer heal up?"

The answer is probably, "Yes." No lowly organized and badly vascularized new tissue could stand the brunt of that inflammation. Probably every hyperplastic epithelial cell has been destroyed and cast off.

And here let us remember that certain portions of the tissue which has been destroyed has been cast off some time before other portions, and that the normal tissue underneath has been subjected to considerable poulticing along with the still undetached portions. Heat and moisture will favor vigorous granulation, and we must beware of mistaking such tissue for remnants of the carcinoma.

It is now safe to let the ulcer heal. It was formerly my custom to use iodoform for that purpose; but the objections to its use, especially around the face, were so patent that I have latterly discontinued it. Aristole, ten grains to the ounce of vaseline, may be employed; but nothing gives better results than simple cold cream, ugt. aq. rosæ, or plain zinc oxide ointment. With this, then, the ulcer is dressed as often daily as may be necessary, depending on the amount of secretion; each dressing being preceded by a careful cleansing of the parts with creolinised warm water.

In a day or two the new tissue begins to shoot inwards from the margins of the ulcer. It closes almost visibly, shrinking day by day. All obstacles to normal repair being removed, healing is very rapid. Even the largest are closed in ten days or two weeks.

Under the pressure of the bandage, excessive growth of granulation tissue soon stops. It may be necessary, however, to touch them with the solid stick.

At length, in the place of the epithelioma, we have a smooth scar, thin and somewhat depressed; of proliferating epithelium there is not a trace.

Our task, however, is not quite completed. The scar must be closely watched, inspected week by week. For it may well happen that in spite of all our care, some little odds and ends of cell nests, or even a single one, may have escaped our destructive inflammation, and start the disease again. This will happen chiefly to those not used to the method of treatment, whom the intensity of the reaction has led to use the escharotic too weak or for too short a time. It happens also in dispensary cases which are not under efficient observation and control. It does not occur if the treatment has been radical.

If then, at the end of four, six, or ten weeks, a small grey nodule appears somewhere in the scar, or if the swelling and induration at the margins of the ulcer does not entirely disappear, or if complete healing does not take place, the treatment must be recommenced. Of course the area now affected is comparatively small. The new nodules and hardened edges must be thoroughly curetted or destroyed with nitric acid, and the arsenical paste reapplied to the spot. It is quite possible that the newly formed scar tissue may break down through the inflammatory process. This is no disadvantage. It will heal again as quickly as before.

Finally, if in four months the scar is found in *statu quo*, growing only whiter and less disfiguring than at first, if the edges where it merges into normal skin are unmarked by any ridges or prominences, traversed by dilated arterioles, we can consider our patient cured.

And if the method herein described is not quick as some others, it is surer, safer, and certainly pleasanter than the more rapid means at our command.

25 West 53d St., New York City.

